# Intravesical Ty21a treatment of non-muscle invasive bladder cancer induces immune responses that correlate with safety and may be associated to therapy potential

**DOI:** 10.1136/jitc-2023-008020

**Published:** 2023-12-14

**Authors:** Laurent Derré, Ilaria Lucca, Valérie Cesson, Perrine Bohner, Francois Crettenand, Sonia-Cristina Rodrigues-Dias, Florence Dartiguenave, Audrey Masnada, Carla Teixeira-Pereira, Sulayman Benmerzoug, Mathieu F Chevalier, Sonia Domingos-Pereira, Sylvain Nguyen, Lenka Polak, Anna K Schneider, Patrice Jichlinski, Beat Roth, Denise Nardelli-Haefliger

**Affiliations:** Urology Research Unit and Urology Biobank, Deptment of Urology, Centre Hospitalier Universitaire Vaudois, Lausanne, Vaud, Switzerland

**Keywords:** Urinary Bladder Neoplasms, Translational Medical Research, Tumor Microenvironment, Immunity

## Abstract

**Background:**

Standard of care treatment of non-muscle invasive bladder cancer (NMIBC) with intravesical Bacillus Calmette Guérin (BCG) is associated with side effects, disease recurrence/progression and supply shortages. We recently showed in a phase I trial (NCT03421236) that intravesical instillation in patients with NMIBC with the maximal tolerated dose of Ty21a/Vivotif, the oral vaccine against typhoid fever, might have a better safety profile. In the present report, we assessed the immunogenicity of intravesical Ty21a in patients of the clinical trial that had received the maximal tolerated dose and compared it with data obtained in patients that had received standard BCG.

**Methods:**

Urinary cytokines and immune cells of patients with NMIBC treated with intravesical instillations of Ty21a (n=13, groups A and F in NCT03421236) or with standard BCG in a concomitant observational study (n=12, UROV1) were determined by Luminex and flow cytometry, respectively. Serum anti-lipopolysaccharide Typhi antibodies and circulating Ty21a-specific T-cell responses were also determined in the Ty21a patients. Multiple comparisons of different paired variables were performed with a mixed-effect analysis, followed by Sidak post-test. Single comparisons were performed with a paired or an unpaired Student’s t-test.

**Results:**

As compared with BCG, Ty21a induced lower levels of inflammatory urinary cytokines, which correlated to the milder adverse events (AEs) observed in Ty21a patients. However, both Ty21a and BCG induced a Th1 tumor environment. Peripheral Ty21a-specific T-cell responses and/or antibodies were observed in most Ty21a patients, pointing the bladder as an efficient local immune inductive site. Besides, Ty21a-mediated stimulation of unconventional Vδ2 T cells was also observed, which turned out more efficient than BCG. Finally, few Ty21a instillations were sufficient for increasing urinary infiltration of dendritic cells and T cells, which were previously associated with therapeutic efficacy in the orthotopic mouse model of NMIBC.

**Conclusions:**

Ty21a immunotherapy of patient with NMIBC is promising with fewer inflammatory cytokines and mild AE, but induction of immune responses with possible antitumor potentials. Future phase II clinical trials are necessary to explore possible efficacy of intravesical Ty21a.

**WHAT IS ALREADY KNOWN ON THIS TOPIC**

Standard of care immunotherapy of non-muscle invasive bladder cancer (NMIBC) with intravesical Bacillus Calmette-Guérin (BCG) is associated with side effects, disease recurrence/progression and supply shortages. We recently showed in a phase I clinical trial in patients with NMIBC that intravesical instillation of Ty21a/Vivotif, the oral vaccine against typhoid fever, might have a better safety profile.

**WHAT THIS STUDY ADDS**

Here we report that Ty21a induced lower levels of urinary inflammatory cytokines than BCG, which correlated to the milder adverse events observed with Ty21a. Nevertheless, robust immune responses were induced by Ty21a including Th1 tumor microenvironment, Ty21a-specific ⍺βT cells and antibodies and activation of Vδ2 T cells. Most importantly, few Ty21a-instillations were sufficient for locally increasing dendritic cells and T cells, which were previously associated to therapeutic efficacy in a mouse model of bladder cancer.

**HOW THIS STUDY MIGHT AFFECT RESEARCH, PRACTICE OR POLICY**

Intravesical Ty21a immunotherapy of NMIBC is thus promising and future phase II clinical trials are warranted to explore its potential efficacy.

## Background

In 2020, an estimated 573,278 new cases of bladder cancer (BCa) occurred worldwide, resulting in 212,536 deaths. This makes BCa the 10th most common malignancy globally.^1^ Seventy per cent of BCas are diagnosed as non-muscle invasive and treated by transurethral resection of the bladder tumor (TURBT).^2^ It is a highly recurrent disease (60–70% of the patients), which when progressing to muscle invasion (10–40% of the patients) requires cystectomy and has a poor outcome.^3^ Beside surveillance by cystoscopy and TURBT, the gold standard treatment for decreasing recurrence/progression of high-grade lesions is intravesical Bacillus Calmette Guérin (BCG) immunotherapy. While the precise mechanisms of action of BCG are still not fully understood, they involve induction of both innate and adaptive immune responses, one of the primary events being infection of the urothelium by the bacteria.^4 5^ BCG then causes a strong local inflammation in the bladder wall including the release of cytokines/chemokines/growth factors.^4 6 7^ An influx of granulocytes and mononuclear cells is then induced together with a Th1 cytokine profile, maturation of dendritic cells (DCs) and activation of natural killer (NK) cells. Although it is the standard treatment to decrease recurrence/progression of non-muscle invasive bladder cancer (NMIBC), repeated BCG treatments (6 weekly instillations and up to 27 maintenance-doses) are associated with significant side effects,^7^ treatment failure in 30–50% of the cases^3^ and frequent shortage,^8^ underlying the necessity for alternative or complementary treatments. We recently tested in a phase I trial (NCT03421236) in patients with low and intermediary risk of progression NMIBC the safety of escalating doses of another bacterial vaccine, the highly attenuated *Salmonella enterica* serovar Typhi strain, Ty21a, administered by the intravesical route.^9^ This strain was obtained almost 50 years ago by mutagenesis^10^ and included in Vivotif, the commercial oral vaccine against typhoid fever. Due to several attenuating mutations, Ty21a has an excellent safety profile confirmed worldwide in more than 200 million vaccinees over the last 30 years.^11^ In the phase I trial (NCT03421236), we reported that intravesical treatment with Ty21a at the maximal tolerated dose (1×10^8^ colony forming units, CFU) in n=13 patients only induced minor adverse events (AEs), no cumulative side effect and lower risks associated to bacterial persistence than with BCG.^9^ In an immunocompetent mouse model, which closely mimics NMIBC in mice,^12^ intravesical Ty21a treatment efficiently induced bladder tumor regression.^13 14^ In contrast to BCG, Ty21a did not persist in mouse and human tissues, was effective at a single dose in absence of strong inflammation and promoted DCs and T-cell-mediated tumor regression, not requiring neutrophils nor NK cells.^13 14^ In the present report, we assessed the immunogenicity of intravesical Ty21a administered at the maximal tolerated dose (groups A and F, n=13 patients) during the phase I trial^9^ and compared with data obtained in patients that had received BCG in a concomitant observational study (n=12 patients, UROV1).

## Methods

### Patients and treatment

Patients with histological confirmation of low or intermediary risk of progression NMIBC, not requiring BCG treatment, received intravesically escalating doses of Ty21a during a phase I clinical trial (NCT03421236), detailed in a study by Lucca *et al*.^9^ In phase Ia, escalating doses of Ty21a were instilled once a week for 4 weeks. The maximal tolerated dose (1×10^8^ CFU, instilled in n=3 patients of group A), was then instilled in phase Ib for 6 weeks in n=10 patient of group F (see characteristics of these patients in online supplemental table S1). Available data, from a non-interventional study in our department (UROV1, approved protocol #2019–00564^15^ with a group of patients with high risk of progression NMIBC (n=12, see characteristics in online supplemental table S2) concurrently treated with intravesical BCG (OncoTICE) during the recruiting period of the Ty21a phase I trial, were used for comparison of urinary cytokines and DCs. These studies were approved by the local state ethics committee and were performed in compliance with the Swiss regulations. All patients provided written informed consent to participate in these studies. *S. enterica* serovar Typhi Ty21a lyophilized bacteria in the format contained in the enteric-coated capsule used for oral immunization (Vivotif, including >2×10^9^ CFU Ty21a) were used within 1 hour after reconstitution and dilution to 1×10^8^ CFU in 50 mL 0.9% NaCl and intravesical instillations were performed like standard BCG treatments. BCG patients (n=12) received six instillations of reconstituted OncoTICE (2–8×10^8^ CFU).

10.1136/jitc-2023-008020.supp1 Supplementary data

10.1136/jitc-2023-008020.supp2 Supplementary data

An AE score to quantify the number and intensity of the AEs observed in Ty21a-treated patients was defined as the sum of the number of AEs × their intensity (ie, 1 for Common Terminology Criteria for Advserse Events (CTCAE) grade 1 or 2 for CTCAE grade 2) at visits 1 to 4 or 6.

### Urinary cytokines

Urine samples collected before (pre) and 2–4 hours after (post) the first, fourth, and sixth Ty21a or BCG instillation were centrifuged at 1500 rpm for 5 min to separate urine supernatant from cells. A panel of cytokines/chemokines/growth factors was measured in cell-free urine collected pre1, post1, pre4, post4, pre6 and post6 Ty21a or BCG instillations using the Bio-Plex Pro Human Cytokine Assay (Bio-Rad) according to manufacture instructions and including GM-CSF (granulocyte-macrophage colony-stimulating factor), G-CSF (Granulocyte colony-stimulating factor), FGF (fibroblast growth factor), VEGF (vascular endothelial growth factor), PDGF-BB (platelet-derived growth factor-BB), eotaxin, MCP-1 (monocyte chemoattractant protein-1), MIP-1α (macrophage inflammatory protein-1α), MIP-1β, RANTES (regulated upon activation, normal T cell expressed and presumably secreted), IP-10 (IFN-gamma-inducible protein 10), interleukin (IL)-2, TNF-α (tumor necrosis factor-α), interferon (IFN)-γ, IL-4, IL-5, IL-6, IL-7, IL-8, IL-9, IL-10, IL-12 (p70), IL-13, IL-15, IL-17A, IL-1β, IL-1RA. An inflammatory score was defined as the sum of fold-changes of pro-inflammatory cytokines/chemokines (MIP-1α, MIP-1β, IL-8, TNF-α, and IL-6) increased on Ty21 instillations. In addition, urinary Th1 (IL-2, TNF-α and IFN-γ) and Th2 (IL-4, IL-5, and IL-13) cytokine concentrations were first normalized to their respective geometric means in (preTy21a1+preBCG1) urinary samples and a Th1/Th2 ratio was then calculated at each time point as (IL-2+TNF-α+IFN-γ)/(IL-4+IL-5+IL-13).

### Specific immune responses

Lipopolysaccharide (LPS)-Typhi specific IgG were determined in serum samples obtained before the first Ty21a instillation and 2 weeks after the fourth (group A) or the sixth instillation (group F). End-point titers (reciprocal of the highest serum dilution that yielded an optical density >0.1) were determined with an ELISA kit from BioVision (E4678-100-1).

Ty21a-specific T cells were determined in peripheral blood monocytic cells (PBMC) collected before Ty21a instillation (V1) and at visit 4 and/or 6. PBMCs were isolated by Isopaque Ficoll density gradient centrifugation and immediately cryopreserved in Roswell Park Memorial Institute (RPMI) supplemented with 40% fetal calf serum (FCS) and 10% dimethyl sulfoxide (DMSO). PBMCs (1×10^6^ cells/well) were then seeded in 48-well plates in antibiotic-free RPMI medium, supplemented with 10% FCS. Cells were then stimulated for 1 hour and 30 min with 1×10^6^ CFU of Ty21a Vivotif (multiplicity of infection, MOI=1, calculated assuming a CFU content=2×10^9^) or left unstimulated in medium alone. After 1 hour and 30 min, Protein Transport Inhibitor Cocktail (eBioscience) was added to each well together with gentamicin (50 µg/mL final), anti-pan TCRγδ PE (11F2) (Beckman Coulter) and anti-CD107a BV605 (H4A3, BioLegend). Cells were incubated for an additional 20 hours at 37°C in 5% CO2. Following incubation, cell-surface antigens were stained for 20 min at 4°C in the staining buffer (phosphate-buffered saline 0.2% bovine serum albumin, 2 mM EDTA), and an amine reactive dye (aqua live/dead stain kit from Life Technologies, Carlsbad, California, USA) was used for dead-cell exclusion according to the manufacturer’s instructions. Fc-Receptor Blocking Reagent (Miltenyi Biotec, Bergisch Gladbach, Germany) was used to increase staining specificity by blocking unwanted binding of antibodies. The following monoclonal antibodies were used at predetermined optimal concentrations: anti-CD4 BUV661 (M-T477) and anti-CD8 BUV395 (G42-8; both from BD Bioscience); anti-CD3 PE/Cy7 (UCHT1) and anti-TCRVδ2 AF700 (B6; both from BioLegend); anti-TCRVδ1 FITC (TS8.2; Thermo Fisher Scientific); anti-pan TCRγδ PE (11F2; Beckman Coulter). For intracellular cytokine labeling, cells were fixed and stained for 30 min at room temperature using Intracellular Fixation and Permeabilization Buffer set (eBioscience), anti-IFN-γ BV421 (4S.B3) and anti-TNF-⍺ AF647 (Mab11; both from BioLegend). When indicated, PBMC from healthy individuals (n=6) were similarly stimulated with Ty21a (Vivotif) or BCG (OncoTICE) both at MOI 0.1 or 1 (calculated with a CFU content of 2×10^9^ for Ty21a and 5×10^8^ for BCG). Sample acquisition was performed on the CytoFLEX LX2 flow cytometer (Beckman Coulter, Brea, California, USA), and data were analyzed using the FlowJo Software (FlowJo LLC, Ashland, Oregon, USA) and SPICE V.6.1^16^ (see gating strategy in online supplemental figure S1).

10.1136/jitc-2023-008020.supp3 Supplementary data

### Urinary immune cell infiltration

The number of cells recovered in urine was independent from the volume collected (data not shown). After Ty21a instillations, when >0.5×10^6^ cells/urine sample were available, CD3^+^ T cells, CD56^+^ NK cells, CD15^+^ neutrophils and CD14^+^ monocytes were determined after antibody staining and flow cytometry analysis (online supplemental figure S2A).^15 17^ The following monoclonal antibodies were used at predetermined optimal concentrations: anti-CD3 PE/AF610 (7D6; from Invitrogen, Thermo Fisher Scientific), anti-CD56 PE (5.1H11), anti-CD15 PerCP/Cy5.5 (W6D3), anti-CD14 Pacific blue (HCD14; all from BioLegend). When >3×10^6^ cell/urine samples were available, a second flow-cytometry panel was used to determine total DCs, conventional DC1 cells (cDC1) and conventional DC2 (cDC2) numbers (online supplemental figure S2B). Such a panel was also used to determine total DCs, cDC1 and cDC2 numbers in available pre2, post2, pre3, post3, pre4, post4 and pre5 and post5 BCG urinary samples. The following monoclonal antibodies were used at predetermined optimal concentrations: anti-CD14 FITC (HCD14), anti-CD141 PE (M80), anti-CD1c PerCP/Cy5.5 (L161), anti-HLA-DR PE/Cy7 (L243), anti-CLEC9a APC (8F9), anti-CD11c BV421 (Bu15; all from BioLegend); anti-CD3 PEAF610 (7D6), anti-CD19 PE/AF610 (SJ25C1), anti-CD56 PE/Texas Red (MEM-188; all from Invitrogen, Thermo Fisher Scientific). Cell-surface antigens were stained as described above. Sample acquisition was performed on the Gallios flow cytometer (Beckman Coulter, Brea, California, USA), and data were analyzed using the FlowJo Software (FlowJo LLC, Ashland, Oregon, USA).

### Statistical analysis

Multiple comparisons of different paired variables were performed with a mixed-effect analysis with the Geisser-Greenhouse correction, followed by Sidak post-test. Single comparisons were performed with a paired or an unpaired Student’s t-test. When data are log_10_ transformed, parametric test was applied, otherwise non-parametric test was used. All statistical analyses were carried out with GraphPad Prism V.9.5.1, except for the polyfunctionality permutation test, which was performed on SPICE V.6.1.A.

## Results

### Urinary cytokines

**Figure 1:**
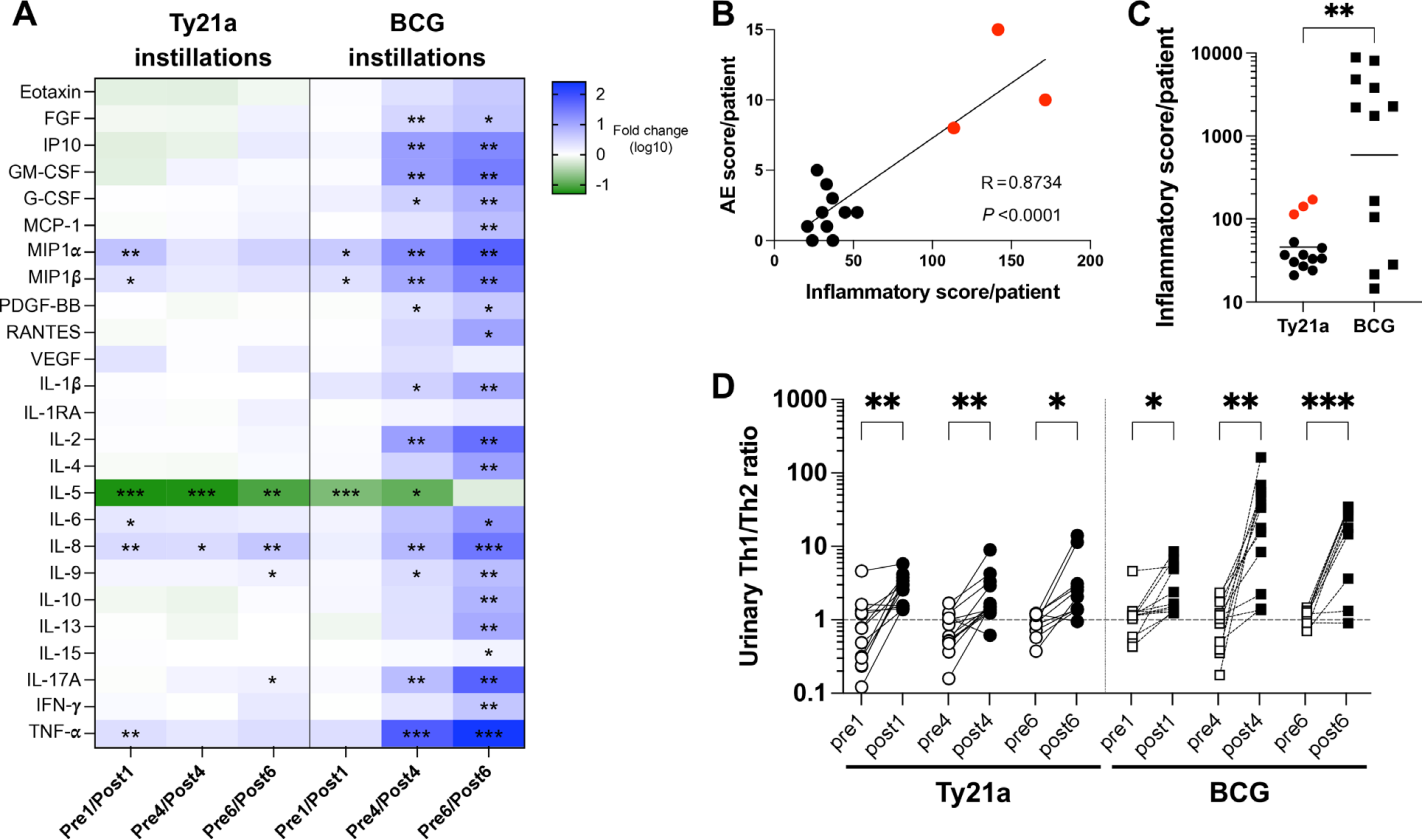
Urinary cytokines and adverse events (AEs) on Ty21a and BCG therapies. (A) Fold pre/post increases of each urinary cytokine, chemokine and or growth factor after Ty21a or BCG instillations are shown as a heat map. Significant fold-increases (as in online supplemental figures S3 and S4) are indicated by *p<0.05, **p<0.01, ***p<0.001. (B) The inflammatory scores of each Ty21a patient (sum at V1, V4 and V6 of the individual fold increases between pre/post samples of the inflammatory cytokines TNF-α, IL-6, IL-8, MIP-1α and MIP-1β on Ty21a) are plotted against the AE score (sum of the number of AEs × their intensity (one for CTCAE grade 1 or 2 for CTCAE grade 2) at each visit V1 to V6, see online supplemental figure S5 of the corresponding patient. Patients with the highest AE score are shown in red. Correlation was assessed by a Pearson test, R and p values are indicated. (C) The inflammatory scores of Ty21a and BCG patients (calculated as in B) are shown. horizontal bars indicate geometric means. Groups were compared by an unpaired standard t-test. **p<0.01. (D) Urinary Th1/Th2 ratio in Ty21a- and BCG-treated patients. Urinary Th1 (IL-2, TNF-α and IFN-γ) and Th2 (IL-4, IL-5, and IL-13) cytokine concentrations were first normalized to their respective geometric means in (preTy21a1+preBCG1) urinary samples. Th1/Th2 ratio was then calculated at each time point as (IL-2+TNF-α+IFN-γ) / (IL-4+IL-5+IL-13) and plotted as paired samples. Significant differences between paired groups are indicated by *=p<0.05, **=p<0.01, and ***=p<0.001. BCG, Bacillus Calmette-Guérin; IFN, interferon; IL, interleukin; TNF, tumor necrosis factor; GM-CSF, granulocyte-macrophage colony-stimulating factor; G-CSF, granulocyte colony-stimulating factor; FGF, fibroblast growth factor; VEGF, vascular endothelial growth factor; PDGF-BB, platelet-derived growth factor-BB; MCP, monocyte chemoattractant protein; MIP, macrophage inflammatory protein; RANTES, regulated upon activation normal T cell expressed and presumably secreted; IP-10, IFN-gamma-inducible protein 10; CTCAE, common Terminology Criteria for Adverse Events.

To assess the extent of inflammation/immune modulation induced by intravesical Ty21a, we measured in urine, before (pre) and after (post) the first, fourth and sixth instillation, a panel of growth factors, chemokines, and cytokines (online supplemental figure S3). Only a few inflammatory cytokines were significantly increased by Ty21a (<5-fold, MIP-1α, MIP-1β, IL-8, TNF-α, and IL-6) (figure 1A). This is in contrast to the large panel of cytokines known to be increased by BCG.^6 18–21^ Thus, to get more insight into the difference between Ty21a and BCG instillations, we took advantage of urinary cytokine data obtained in a parallel study undergoing in our hospital (online supplemental figure S4) and (online supplemental table S2). Despite of the difference in patients with NMIBC, high-risk of progression for BCG (online supplemental table S2) and low/intermediary risk for Ty21a (online supplemental table S1 and 9), the levels of the cytokines at baseline were not significantly different in the two groups of patients, except for IL-6 and VEGF (ca. twofold higher in pre BCG1 vs preTy21a1, data not shown). Comparison of the geometric mean cytokine fold-increase in each group of patients (figure 1A), confirmed the greater effect of BCG,^6 18–21^ especially after the fourth treatment, with several inflammatory cytokines increased >20-fold. Interestingly, the increases of urinary inflammatory cytokines, expressed as an inflammatory score, in individual Ty21a patients was strongly correlated to the number and intensity of the AEs they experienced (figure 1B and online supplemental figures S5 and S6), with only 3/13 patients with an AE score >5 (online supplemental figure S6). Comparison between Ty21a and BCG patients showed that 7/12 BCG-treated patients had an inflammatory score >10-fold higher than any of the Ty21a-patients (figure 1C), in agreement with the important side effects generally observed in BCG patients.^7^

Furthermore, the most modulated analyte on Ty21a installations is IL-5, a Th2 cytokine, as it decreased >10-fold after each instillation. BCG also significantly decreased IL-5 after the first and fourth instillation, but to a lesser extent (6-fold to 8-fold) than Ty21a (18-fold). In addition, BCG also increased ca.10-fold the Th2 cytokines IL-4 and IL-13, while Ty21a did not significantly increased these cytokines, suggesting that Ty21a might maintain a Th1/Th2 balance in favor of Th1, despite of the lower effect on Th1 cytokines (figure 1A). Thus, calculation of a Th1/Th2 ratio (including the Th1 cytokines TNF-α, IFN-γ, IL-2 and the Th2 cytokines IL-4, IL-5 and IL-13, figure 1D), confirmed that, similarly to BCG, each Ty21a instillation significantly increased the Th1/Th2 ratio towards a Th1 microenvironment into the bladder.

### Ty21a-specific immune responses

To get insight into a possible specific immune response to Ty21a, we measured the presence of anti-LPS Typhi IgG in serum before and after Ty21a treatment (table 1).

**Table 1:** Anti-lipopolysaccharide Typhi IgG serum titer

Patients	Serum titers*	
	Pre Ty21a instillation	2 weeks post fourth or sixth instillation
1A01	<50	200†
3A02	<50	<50
4A03	<50	<50
9F01	50	50
11F02	100	800
12F03	50	200
14F04	50	<50
17F05	<50	800
19F06	<50	800
22F07	200	1600
23F08	<50	<50
24F09	50	50
25F10	50	100

*End-point titers, reciprocal of the highest dilution that yielded an optical density>0.1.

†Seroconverted patients are shown in bold (≥4-fold increase between pre and post samples).

**Figure 2:**
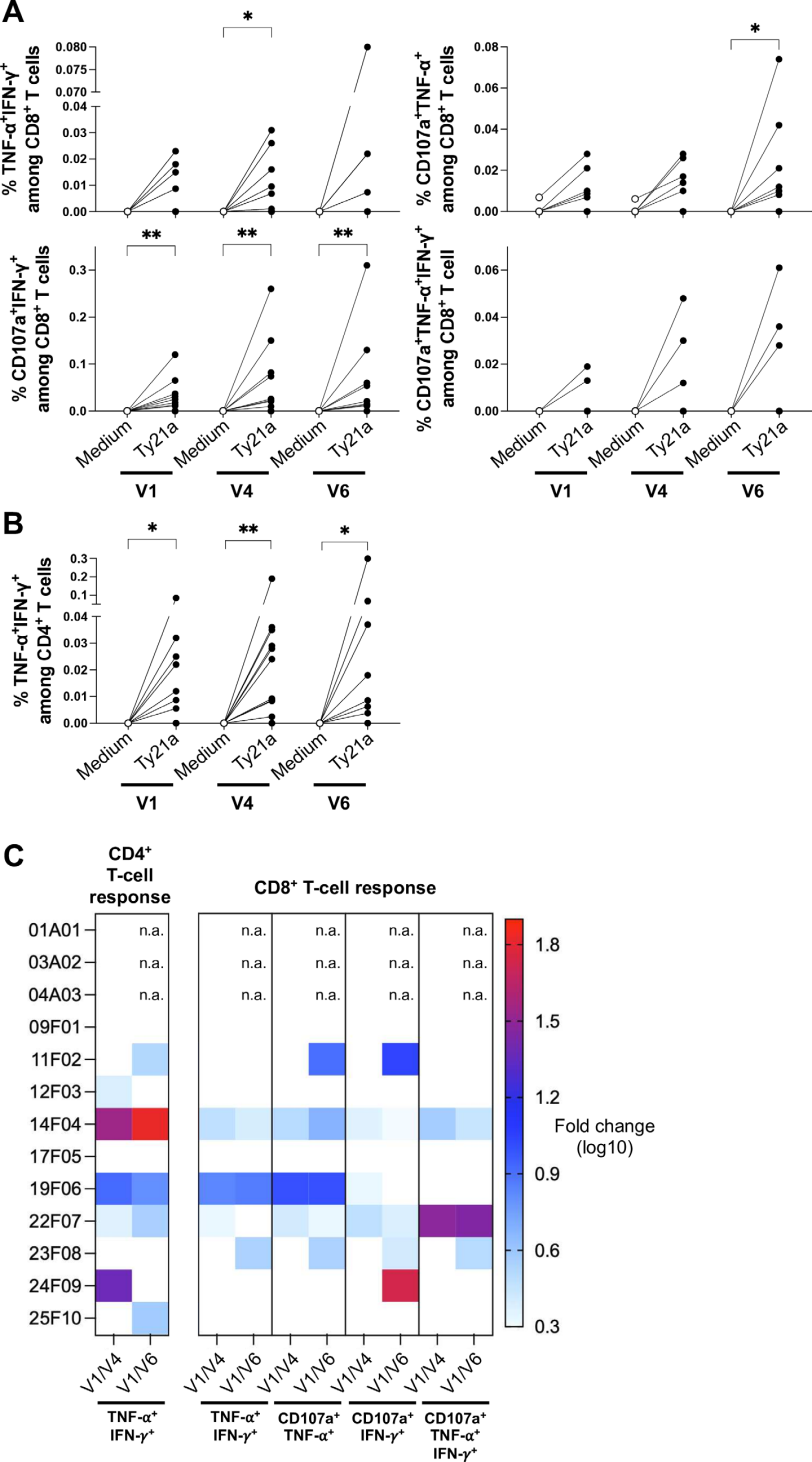
Circulating Ty21a-specific T-cell responses. Peripheral blood monocytic cells of patients (n=13) at visit 1 (V1), visit 4 (V4) and visit 6 (V6, only group F, n=10) were cultured in medium alone or in presence of Ty21a bacteria (multiplicity of infection=1). Percentages of (A) TNF-α^+^IFN-γ^+^, CD107a^+^TNF-α^+^, CD107a^+^IFN-γ^+^, or CD107a^+^IFN-γ^+^TNF-α^+^ among CD8^+^ T cells and (B) TNF-α^+^IFN-γ^+^CD4^+^ T cells. (C) Heatmap of the Ty21a-specific T-cell response fold (>2-fold) increase (between V1/V4 and V1/V6) on Ty21a treatment per patient. n.a. not applicable. Significant differences between paired groups are indicated by *=p<0.05, **=p<0.01. IFN, interferon.

The data showed seroconversion (≥4-fold increase in titers) in 6/13 patients, suggesting that intravesical Ty21a bacteria were sensed by the adaptive immune system locally in the bladder of these patients. We next tested whether Ty21a-specific T-cell responses were also induced. For this purpose, PBMC obtained before the first (V1), fourth (V4) and sixth (V6, only group F) Ty21a instillation were stimulated in vitro with Ty21a bacteria (MOI=1) and TNF-α, IFN-γ and CD107a double production were determined by flow cytometry (figure 2 and online supplemental figure S1). To be more specific, only double, and triple positive T cells were considered. Thus, double CD107a^+^IFN-γ+CD8^+^ (figure 2A) and TNF-α^+^IFN-γ+CD4^+^ (figure 2B) T cells were significantly induced by Ty21a at all time points, while CD107a^+^CD4^+^ T cells were not detected (data not shown). TNF-α^+^IFN-γ+ and CD107a^+^TNF-α^+^ CD8^+^ T cells were less consistently induced (figure 2A), though triple positive CD8^+^ T cells were detected in three patients (F04, F07 and F08, figure 2A). An increase of these Ty21a-specific CD4^+^ and/or CD8^+^ T cells (>2-fold, ie, 0.3 on the log_10_ scale in the figure 2C) were also observed upon Ty21a treatment in 5/13 patients at V4 and in 7/13 patients at V6. Considering LPS-Typhi seroconversion and/or Ty21a-specific CD4^+^ and CD8^+^ T cells, most patients (10/13) showed an adaptive immune response on Ty21a treatment (no response detected for A02, A03 and F01 patients), confirming the ability of Ty21a bacteria to induce immune responses locally in the bladder.

**Figure 3:**
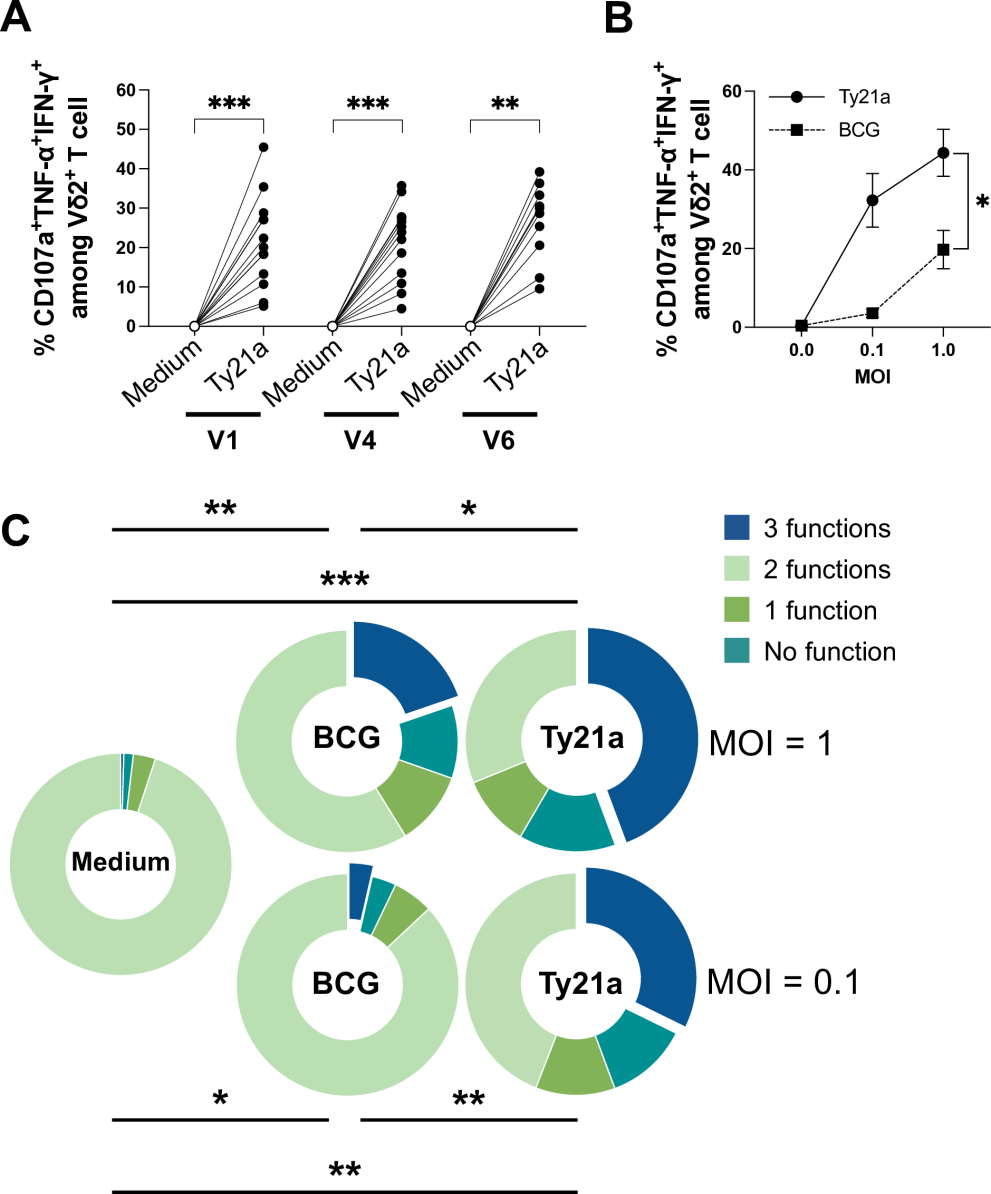
Vδ2 T-cell response against Ty21a. (A) Percentages of CD107a^+^IFN-γ^+^TNF-α^+^ among Vδ2 T cells, after Ty21a stimulation of PBMC from patients (n=13) at visit 1 (V1), visit 4 (V4) and visit 6 (V6, only group F, n=10). PBMC of healthy individuals were cultured overnight with BCG or Ty21a at MOI: 0, 0.1 or 1. (B) Percentages of CD107a^+^IFN-γ^+^TNF-α^+^ among Vδ2 T cells. Area under the curve comparison were used for statistical analysis. (C) Vδ2 T-cell polyfunctionality. Significant differences are indicated by *=p<0.05, **=p<0.01 and ***=p<0.001. BCG, Bacillus Calmette Guérin; IFN, interferon; PBMC, peripheral blood monocytic cells; MOI, multiplicity of infection, TNF; tumor necrosis factor.

Beside conventional αβ CD4^+^ and CD8^+^ T cells, we surprisingly noticed another population of circulating T cells that was strongly reacting to Ty21a in vitro, namely the Vδ2 subset of the unconventional γδ T cells (online supplemental figure S1B). It has been shown that Vδ2 T cells, can be activated by BCG and may play a role in bladder tumor control.^22 23^ Activation of Vδ2 T cells by Ty21a in vitro was observed for all the patients, with induction of 10–50% triple positive TNF-α^+^IFN-γ+CD107a^+^ effector cells (figure 3A). Of note, no Vδ1 T-cell reactivity was found (data not shown). To get more insights into the ability of Ty21a to activate Vδ2 T cells, we performed a comparison with BCG by infecting PBMC from healthy donors (n=6). Activation by Ty21a turned out to be significantly more efficient, inducing more triple positive TNF-α^+^IFN-γ+CD107a^+^ (figure 3B) and polyfunctional effector Vδ2 T cells (figure 3C) than BCG. The declared, relatively variable, CFU contents of both the clinical products Ty21a/Vivotif (>2×10^9^) and BCG (3–8×10^8^) may possibly influence MOI comparisons. However, Ty21a resulted significantly more efficient than BCG to stimulate Vδ2 T cells even at a calculated 10-fold lower MOI (p=0.002 for BCG MOI 1 vs Ty21a MOI 0.1 for polyfunctionality in figure 3C, and p=0.0312 in online supplemental figure S7 for triple positivity). The powerful and rapid activation obtained in vitro, Ty21a extracellular bacteria being killed by gentamycin after 1 hour 30 min, raise the possibility that intravesical Ty21a might also activate Vδ2 T cells locally in the bladder.

### Urinary immune-cell infiltration

**Figure 4:**
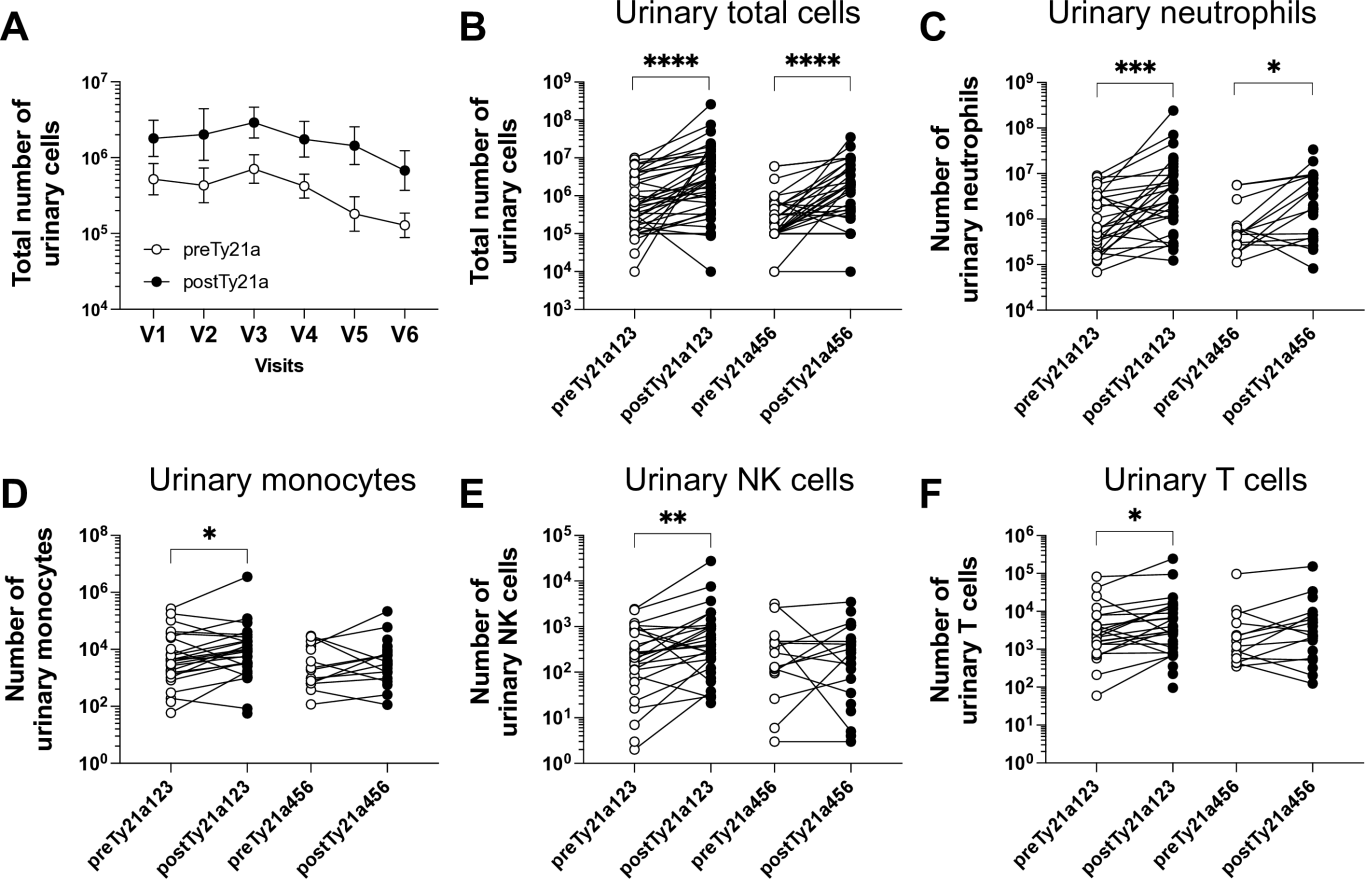
Immune cells infiltration in urine along Ty21a treatment. (A) Absolute number of urinary cells from patients of group A and F, before and after each Ty21a instillation were recorded and geometric mean values at each visit (V1 to V6) are shown (preTy21a, white circle; postTy21a, black circle). (B) Number of urinary cells in individual patients obtained before and after instillations are shown as paired samples between pre1, 2 and 3 versus post1, 2 and 3 instillations, as well as between pre4, 5, and 6 versus post4, 5 and 6 instillations. When enough number of urinary cells were available number of neutrophils (C) monocytes (D) NK cells (E) and T cells (F) were determined by flow cytometry. A connecting line indicates paired pre/post samples. Significant differences between paired groups are indicated by *=p<0.05, **=p<0.01, ***=p<0.001 and ****=p<0.0001. NK, natural killer.

**Figure 5:**
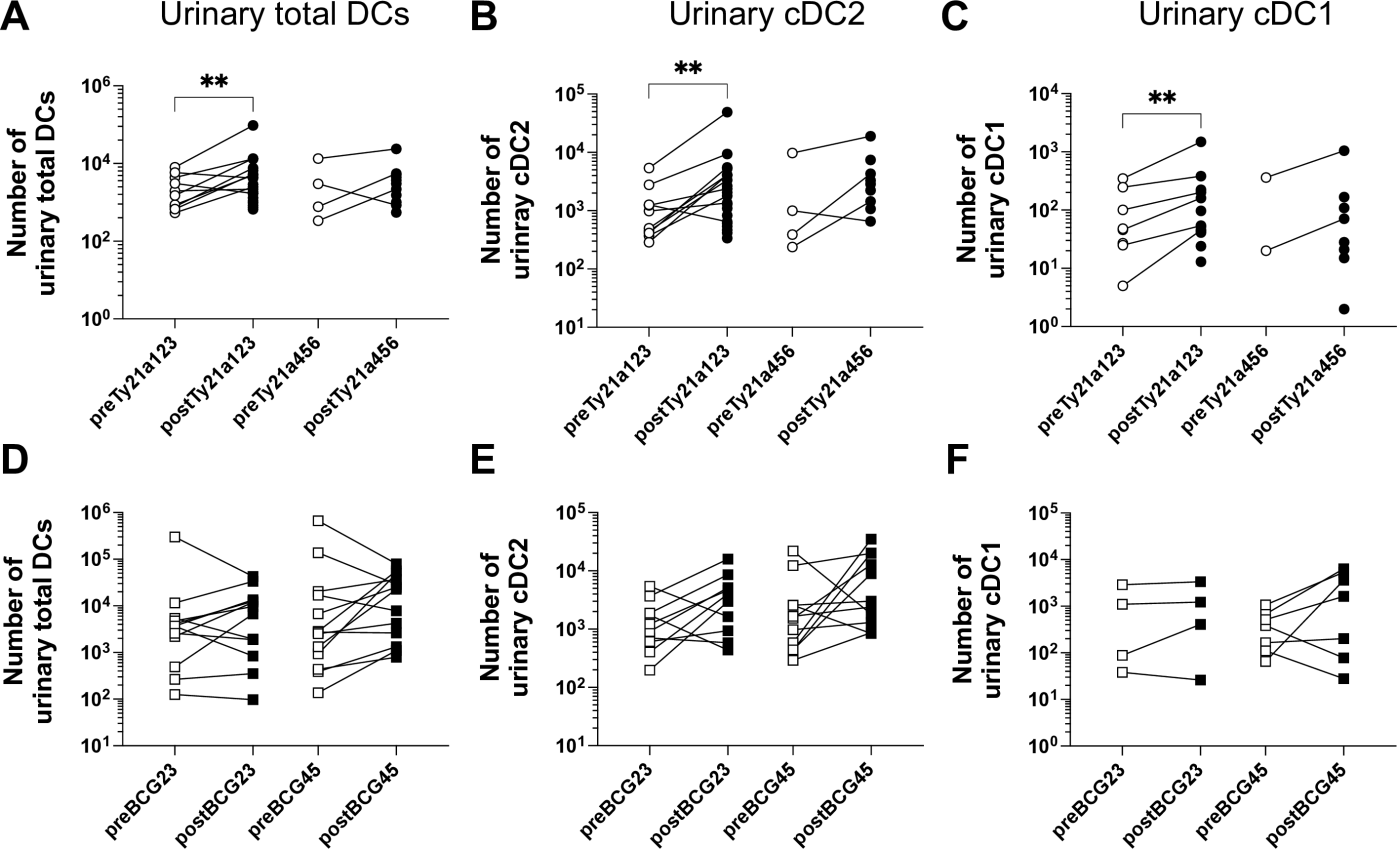
Urinary DC infiltration along Ty21a or BCG treatments. When enough urinary cells were available numbers of total DCs (A and D) cDC2 (B and E) and cDC2 (C and F) were determined by flow cytometry after Ty21a (A–C) or BCG (D–F) treatments. Connecting line indicates paired pre/post samples. Significant differences between paired groups are indicated by **=p<0.001. BCG, Bacillus Calmette Guérin; cDC1, conventional DC1 cells; cDC2, conventional DC 2 cells; DC, dendritic cell.

To gain a deeper understanding of immune responses locally induced by Ty21a, we assessed urinary immune cell infiltration along Ty21a immunotherapy. We observed that overall, the total number of urinary cells tends to increase at each Ty21a instillations (figure 4A). The individual numbers of urinary cells recovered pre or postTy21a was highly variable between patients and samples before and along the Ty21a treatment. Because of the paucity of cells recovered in some of the urinary samples (particularly before each Ty21a instillation, and after the fourth treatment), we considered for statistical analysis pre1, pre2 and pre3 samples together (preTy21a123) as well as pre4, pre5 and pre6 samples together (preTy21a456) and their counterpart post samples. A significant increase in total infiltrating cells and neutrophils (figure 4B–C), which represent >80% of urinary immune cell subsets (data not shown), was observed between paired preTy21a123 and postTy21a123 samples, as well as between paired preTy21a456 and postTy21a456 samples (figure 4B–C); cell infiltration was similar between preTy21a123 and preTy21a456 or between postTy21a123 and postTy21a456 (online supplemental figure S8A). This is significantly different from results obtained after BCG instillation were number of urinary cells are increasing along the treatment (online supplemental figure S8B), in agreement with previous reports.^6 24^ Moreover, monocytes, NK and T cells were significantly increased only during the first half of the Ty21a therapy (figure 4D–F). Since DCs and particularly cross-presenting DCs were shown to be associated to therapeutic efficacy of Ty21a intravesical instillations in a bladder tumor mouse model,^14^ we also determined urinary DC infiltration in Ty21a patients and, in absence of thorough DCs data after intravesical BCG,^25 26^ compared with available urinary samples (pre2 to post5 BCG) from the parallel UROV1 study. We found that in contrast to BCG, Ty21a intravesical immunotherapy induces a significant increase of urinary total DCs, cDC2 (CD1c^+^) and cDC1 (CLEC9a^+^CD141^+^) in paired preTy21a123/postTy21a123 samples (figure 5), suggesting that DCs increase may be more an attribute of Ty21a.

## Discussion and conclusion

Despite regained interest in microbial cancer immunotherapy, intravesical BCG, limiting recurrence/progression of NMIBC, is still the unique bacterial cancer therapy approved for clinical use. Building on our promising preclinical data,^13 14^ here we show that another commercialized bacterial vaccine, Ty21a/Vivotif, used as intravesical instillations in patients with NMIBC, has not only a good safety profile (online supplemental figures S5 and S9), but is also immunogenic resulting in a Th1 bladder microenvironment in absence of strong inflammation, infiltration of DCs and T cells that might participate in tumor control together with Ty21a-specific CD8^+^ and CD4^+^ T cells and activated Vδ2 T cells.

For ethical reason, Ty21a treatment was assessed in patients with NMIBC not requiring the standard BCG therapy (ie, low/intermediary risk of progression patients), but in conditions mimicking at most the use of BCG (ie, 6 weekly intravesical instillations starting 3–4 weeks after TURBT). Indeed, the levels of urinary analytes was similar in our two groups of patients before treatment allowing subsequent comparison. Interestingly, our data showed that after Ty21a treatment the urinary levels of a set of inflammatory cytokines (TNF-α, IL-6, IL-8, MIP-1α, and MIP-1β) were all correlating to the AEs experienced by the patients (figure 1B and online supplemental figure S6A). These cytokines are known to induce inflammatory reactions and pain in the peripheral and central nervous system^27^ and were associated to bladder conditions in patients with bladder pain syndrome and interstitial cystitis,^28^ but to our knowledge not previously associated to side effects in the case of intravesical treatments, including BCG. As compared with Ty21a patients, the 10-fold higher levels of these cytokines in 60% of our BCG patients, is possibly predictive of the known high reactogenicity of BCG treatment,^7^ although this was not examined in our BCG cohort. Of note, the kinetic of inflammatory cytokine induction was also quite different between Ty21a, with only a significant induction at the first instillation (except for IL-8), and BCG, with higher fold-increases after the fourth and sixth instillations (figure 1A). A phenomenon, we already observed in the mouse model^13 14^ and which may relates to previous reports on exposure to LPS of gram-negative bacteria (like Ty21a) transiently silencing inflammatory cytokines to prevent excessive inflammation.^29 30^ This is also in agreement with the observation that AEs after Ty21a were limited to few instillations with no cumulative side effects along treatment,^9^ in contrast to BCG.^31 32^ Intravesical Ty21a induced less inflammatory and Th1 cytokines than BCG (figure 1A), but nevertheless resulted in a Th1 anti-tumorigenic microenvironment (reviewed in^33^) because Th2 cytokines were poorly enhanced (figure 1A). Strikingly a Th2 cytokine, IL-5, was even strongly decreased in urine on Ty21a, and to a lesser extent BCG, instillations. IL-5 induces proliferation and maturation of eosinophils,^34^ which were mainly involved in allergic disease and worm infections.^35^ Although, in the lung, BCG decreased IL-5 and suppressed allergen-induced eosinophilia and asthma after intranasal administration in mice,^36^ only one report showed IL-5 increase between post BCG urinary samples.^37^ IL-5 and eosinophils may also create a protumorigenic environment, promoting lung metastasis.^38^ In addition, high levels of IL-5 and eosinophils were associated with migration and invasion of BCa cells^39^ and recurrence of NMIBC,^40^ respectively. Whether decreasing IL-5 participates in Ty21a or BCG immunotherapy of NMIBC remains an open question.

Although the bladder is a rather poor mucosal inductive site, both antigen-specific antibodies and T-cell peripheral responses can be induced after urinary bacterial infections,^41^ as well as BCG-specific T cells after intravesical BCG treatment.^42^ Robust Ty21a-specific immune responses were relatively unexpected as, in contrast to BCG, Ty21a bacteria after intravesical instillations did not persist in bladder, nor invade deeper organs in mice; and was only detected in 3/72 urinary samples (from F03, F06 and F08 patients) in our cohort of patients.^9^ Nevertheless, Ty21-specific effector T cells were induced in 80% of the patients receiving six intravesical instillations (8/10 in group F), similarly to data obtained after oral vaccination (43and reference therein), revealing the bladder as a more efficient immune inductive site than anticipated. It has been proposed that BCG-specific T cells may attack tumor cells presenting BCG antigen (reviewed in^44^) and induction of BCG-specific immune responses during BCG treatment of BCa was associated with an improved clinical response.^45^ Whether Ty21a-specific T cells may play a role on Ty21a treatment deserve further investigations.

Unconventional γδ T cells also possess cytotoxic properties and can recognize tumor antigens in a major histocompatibility unrestricted manner.^46^ Among The γδ T cells, Vδ2 T-cell subset, another known player in BCG immunotherapy of BCa,^22 23^ can sense non-peptidic phosphorylated antigens, that can be overexpressed by transformed cells, but also produced by BCG.^47^ Our data show that Ty21a can also activate in vitro effector Vδ2 T cells from PBMC in our cohort. (E)−4-hydroxy-3-methyl-but-2-enyl pyrophosphate (HMBPP) has been identified as a microbial metabolite in the 2-C-methyl-D-erythritol-4 phosphate pathway for isoprenoid biosynthesis that is used by many bacteria and protozoan parasites and which is preferentially recognized by human Vδ2 T cells.^48^ Although Salmonella are not commonly listed as Vδ2 T-cell activators, expansion of Vδ2 T cells by *Salmonella typhi* and typhimurium in vitro and in vivo was reported.^49^ In addition, engineering of a *Salmonella typhimurium* vaccine strain deleted in the *LytB* gene, thus allowing synthesis of higher amounts of HMBPP, was shown to induce stronger Vδ2 T-cell proliferation.^50^ HMBPP is thus probably the Ty21a microbial metabolite responsible of activation of Vδ2 T cells. Unexpectedly, we observed that Ty21a was a more potent activator of Vδ2 T cells than BCG, suggesting higher HMBPP content in the former. The strong activation by Ty21a observed in vitro suggests that this may also occur locally in the bladder. Considering the role of Vδ2 T cells in tumor control during BCG treatment,^22 23^ Vδ2T cells investigations are warranted in future clinical trials with Ty21a.

In absence of bladder biopsies after the Ty21a therapy, immune infiltration in the urine may be representative of the situation in the urothelium/bladder wall. Analysis of the urinary cells recovered during the first three intravesical Ty21a instillations, demonstrated the ability of Ty21 to induce infiltration of neutrophils, monocytes, NK cells and T cells similarly to BCG,^6^ while DCs were additionally increased by Ty21a, but not by BCG, even during the last instillations (figure 5). In the mouse model, tumor regression upon Ty21a, was mediated by T cells and DCs,^14^ whether this may also be the case in patients with NMIBC will have to wait future clinical trials.

Beside bacterial immunotherapies, several new agents, including gene therapy (eg, Adstiladrin), immune modulators (eg, N-803) and chemotherapies (eg, gemcitabine) are under development and tested as monotherapy or in combination with BCG in patients with NMIBC (reviewed in a study by Shore *et al* 51). Similarly, combinatorial therapies including Ty21a might also be considered.

Although limited by the relatively low numbers of patients included and of urinary cells recovered after the fourth Ty21a treatment, our study shows that Ty21a immunotherapy of patients with NMIBC is promising with a mild inflammation and induction of immune responses with possible antitumor potentials. As this study was a phase I trial focusing on the safety aspect, it only lasted until 2 weeks after the last Ty21a intravesical treatment and did not include a clinical follow-up. Thus, future phase II clinical trials will be necessary to explore the potential efficacy of intravesical Ty21a to reduce recurrence/progression of NMIBC and explore underlying mechanisms.

## Data Availability

Data are available upon reasonable request.
