# Supplementary material for: Intravesical Ty21a treatment of non-muscle invasive bladder cancer induces immune responses that correlate with safety and may be associated to therapy potential
Source: J Immunother Cancer. 2023 Dec 14;11(12):e008020. doi: 10.1136/jitc-2023-008020 (PMC10729085; doi:10.1136/jitc-2023-008020)
Supplement: Supplementary data [file jitc-2023-008020supp001.pdf]

Supplementary Table 1: Patient characteristics of Ty21a-treated patients

Characteristics	Phase Ia (A) 1 x 10^8 CFU[a]	Phase Ib (F) 5 x 10^8 CFU[a]	Total
Number of patients	3	10	13
Age, yr, median (IQR)	76 (71-79)	74 (58-75)	74 (61-76)
**Sex, n**			
Male	3	9	12
Female	0	1	1
**Number of tumors, n**			
Single	2	4	6
2-7	1	6	7
> 8	0	0	0
**Tumor diameter, n**			
< 3 cm	3	9	12
> 3 cm	0	1	1
**Prior recurrence,**			
Primary	2	5	7
< 1 recurrence/yr	1	2	3
> 1 recurrence/yr	0	3	3
**Category and grade, n**			
Ta low grade	3	10	13
Ta high grade	0	0	0
T1 high grade	0	0	0
Any CIS positive	0	0	0
**Risk of recurrence[b], n**			
Low	1	1	2
Intermediate	2	9	11
High	0	0	0
**Risk of progression[b], n**			
Low	1	1	2
Intermediate	2	9	11
High	0	0	0

[a] Data from Lucca et al. Eur Urol Open Sci. 2022 Oct 3;45:55-58.
[b] according to EORTC score
CIS = Carcinoma In Situ; IQR: Interquartile Range
