# Supplementary material for: Intravesical Ty21a treatment of non-muscle invasive bladder cancer induces immune responses that correlate with safety and may be associated to therapy potential
Source: J Immunother Cancer. 2023 Dec 14;11(12):e008020. doi: 10.1136/jitc-2023-008020 (PMC10729085; doi:10.1136/jitc-2023-008020)
Supplement: Supplementary data [file jitc-2023-008020supp002.pdf]

Supplementary Table 2: Characteristics of patients treated by BCG

Characteristics	BCG Tice
Number of patients	12
Age, yr, median (IQR)	66 (53-72)
**Sex, n**	
Male	12
Female	0
**Number of tumors, n**	
1	1
2-7	11
> 8	0
**Tumor diameter, n**	
< 3 cm	9
> 3 cm	3
**Prior recurrence, n**	
Primary	10
< 1 recurrence/yr	1
> 1 recurrence/yr	1
**Stage and grade, n**	
Ta low grade	1
Ta high grade	3
T1 high grade	8
Any CIS positive	6
**Risk of recurrence[a], n**	
Low	0
Intermediate	11
High	1
**Risk of progression[a], n**	
Low	0
Intermediate	0
High	12

CIS = Carcinoma In Situ; IQR = Interquartile Range
[a] according to EORTC score
