# Supplementary material for: Intravesical Ty21a treatment of non-muscle invasive bladder cancer induces immune responses that correlate with safety and may be associated to therapy potential
Source: J Immunother Cancer. 2023 Dec 14;11(12):e008020. doi: 10.1136/jitc-2023-008020 (PMC10729085; doi:10.1136/jitc-2023-008020)
Supplement: Supplementary data [file jitc-2023-008020supp003.pdf]

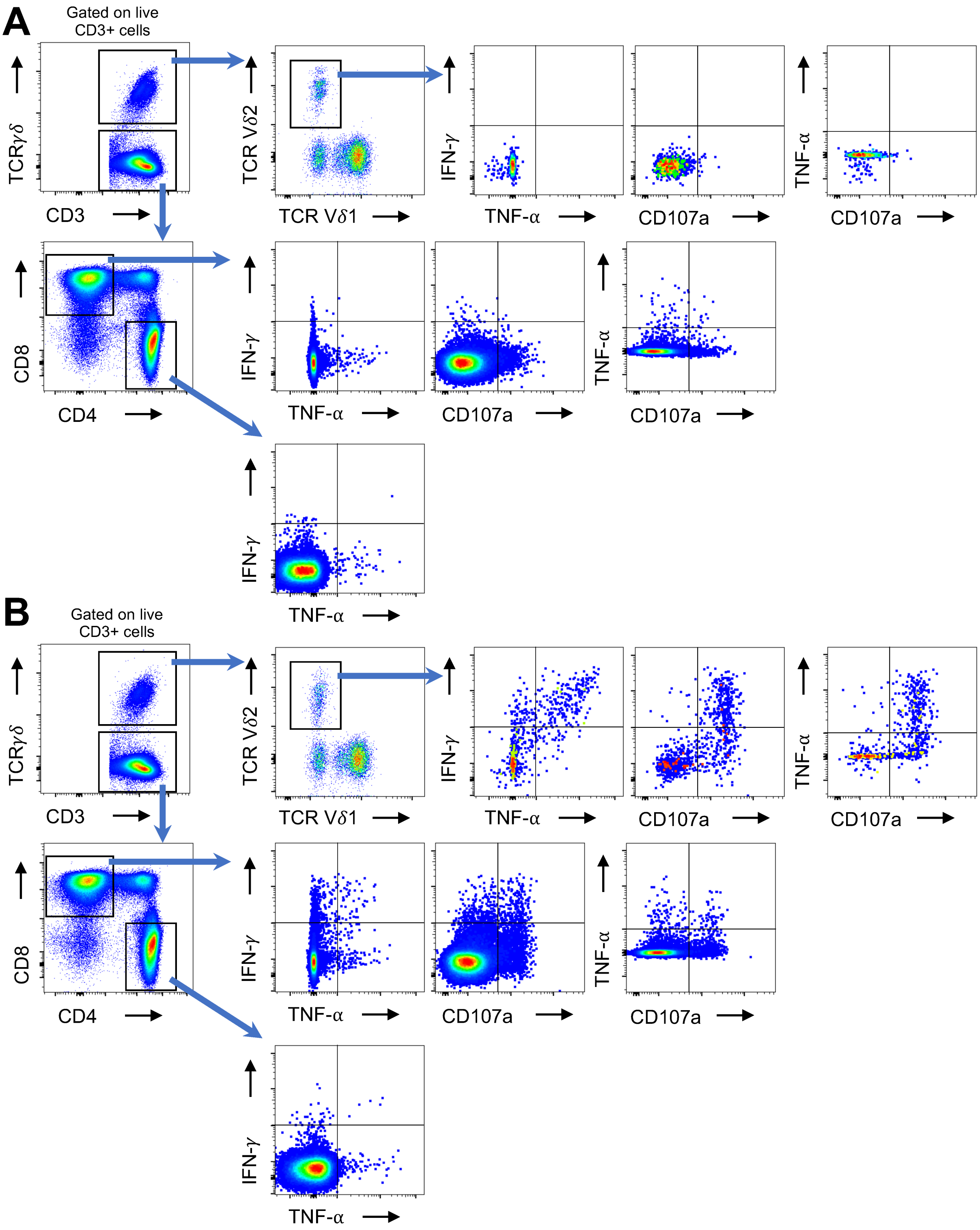

Supplementary Figure S1: Flow cytometry analysis of Ty21a specific T cells

Representative flow cytometry dot plots showing the gating strategy to identify conventional $CD3^{+}CD8^{+}$ and $CD3^{+}CD4^{+}$ T cells and Vδ2 T cells expressing INF-γ and TNF-α, or INF-γ and CD107a or TNF-α and CD107a upon culture **(A)** in medium alone or **(B)** in presence of Ty21a.

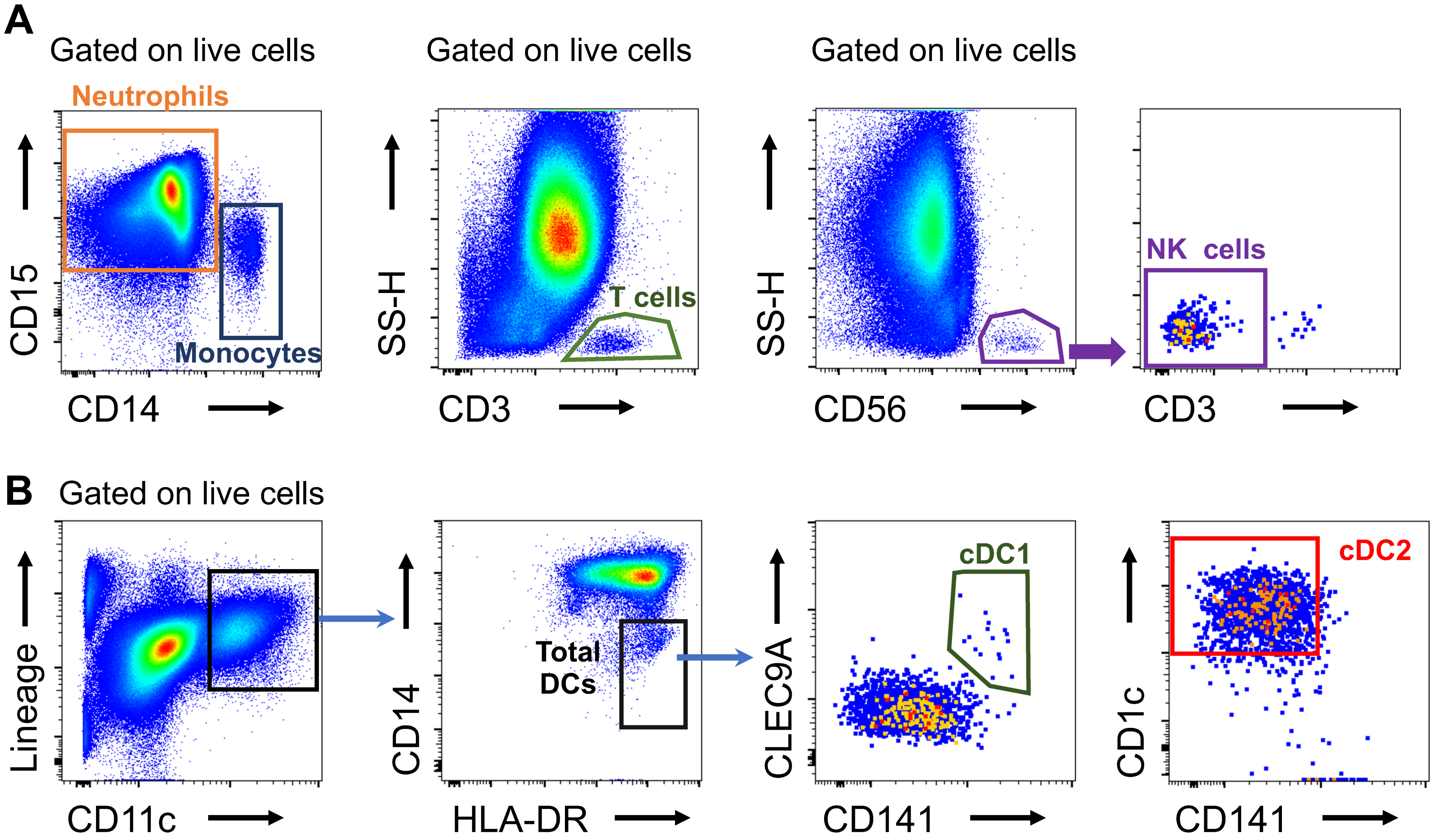

Supplementary Figure S2: Gating strategy to identify urinary immune cell subsets

A, Gating strategy identifying neutrophils ($CD15^{+}CD14^{neg}$), monocytes ($CD15^{neg}CD14^{+}$), T cells ($CD3^{+}$) and NK cells ($CD56^{+}CD3^{neg}$), after dead cell exclusion.
B, Gating strategy identifying dendritic cell subsets: After dead cell exclusion, when gating on lineage-negative (i.e., $CD3^{neg}CD56^{neg}CD19^{neg}$) cells, total dendritic cell (DC) were identified as $CD11c^{+}CD14^{neg}$HLA-DR^{high}. Then from total DC, cDC1 and cDC2 subpopulations were characterized as $CLEC9A^{+}CD141^{+}$ and $CD1c^{+}CD141^{neg}$, respectively.

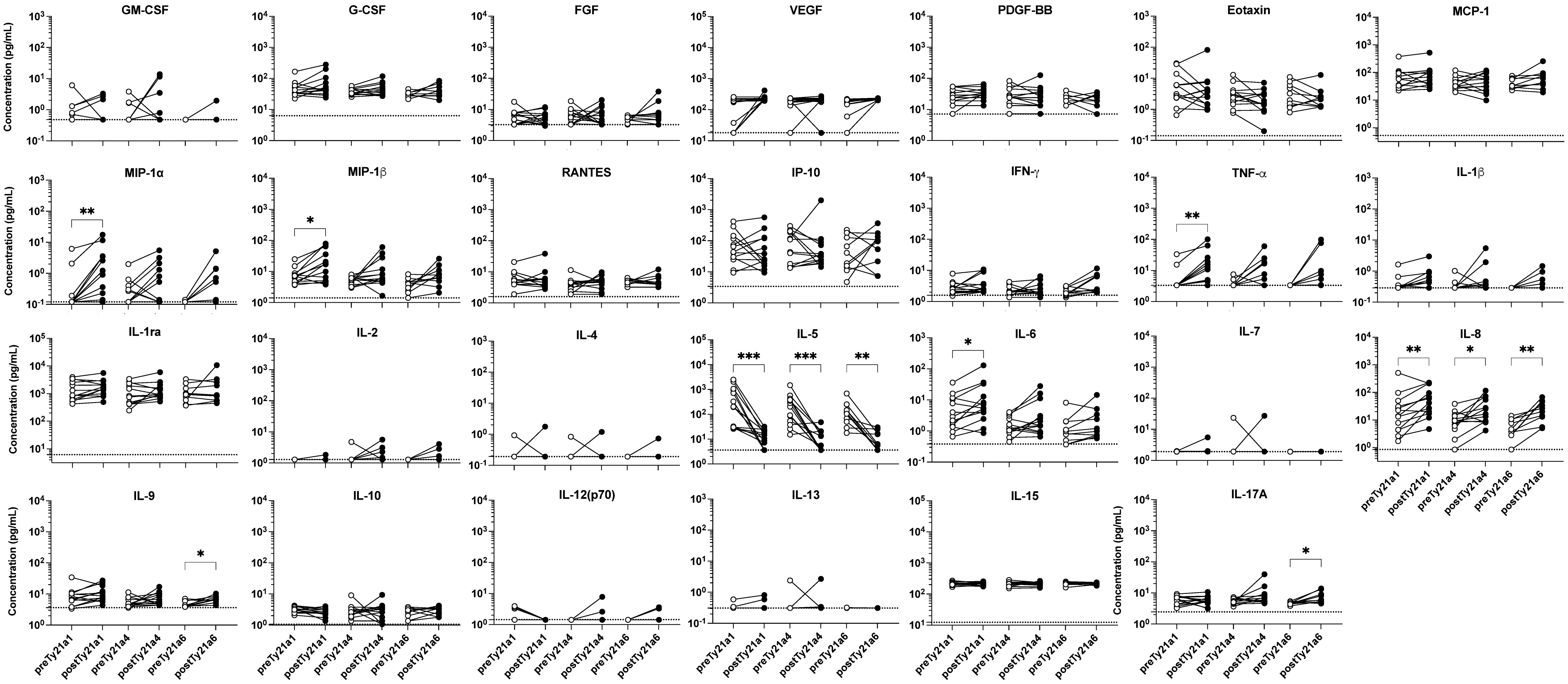

Supplementary Figure S3: Urinary cytokines along intravesical Ty21a treatment

Each cytokine (pg/ml) measured in urinary samples before (white circle) the 1st (preTy21a1), 4th (preTy21a4) and 6th (preTy21a6) instillations, as well as 2-4 hours after those same instillations (black circle, postTy21a1, postTy21a4 and postTy21a6) are plotted as paired samples. Dotted lines represent the limit of detection. Significant differences between paired groups for each cytokine are indicated by * = p < 0.05, ** = p < 0.01, and *** = p < 0.001.

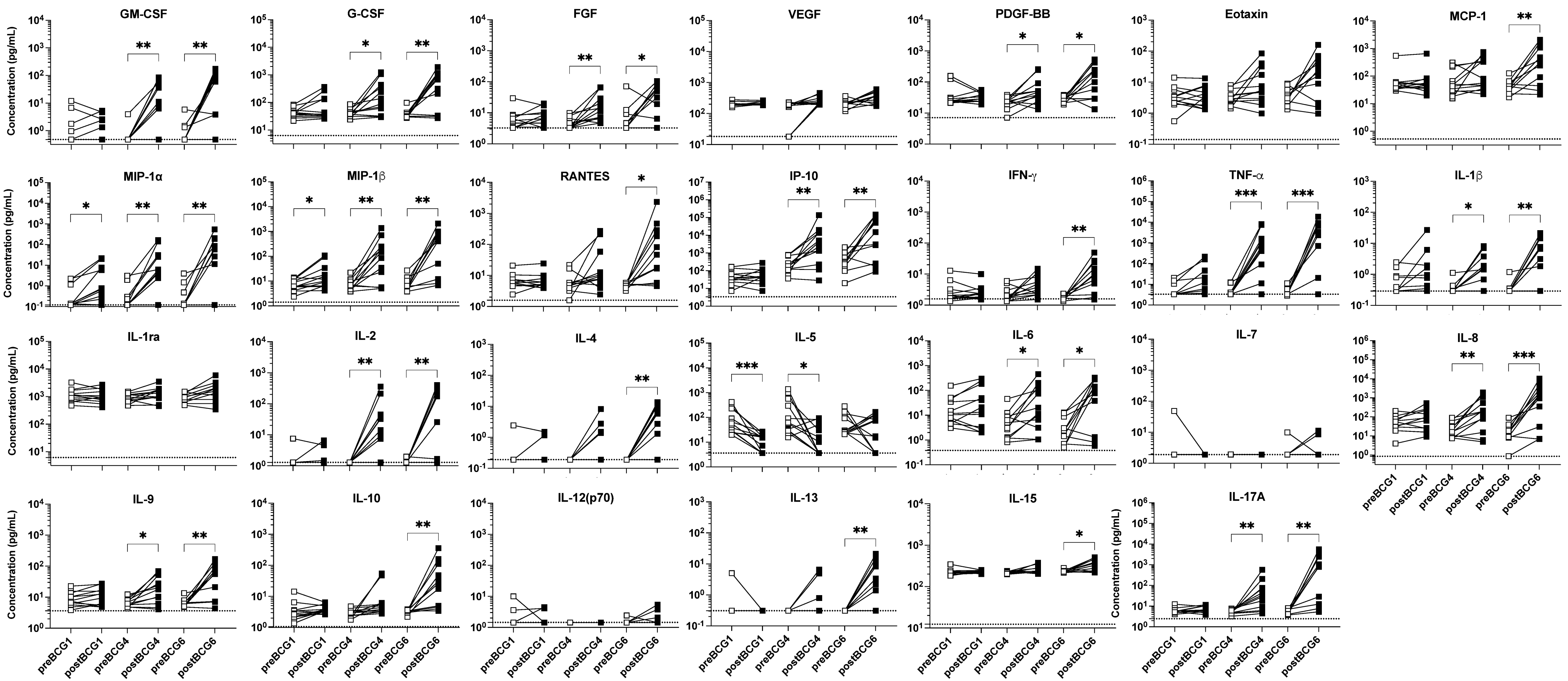

Supplementary Figure S4: Urinary cytokines along intravesical BCG treatment

Each cytokine (pg/ml) measured in urinary samples before (white square) the 1st (preBCG1), 4th (preBCG4) and 6th (preBCG6) instillations, as well as 2-4 hours after those same instillations (black square, postBCG1, postBCG4 and postBCG6) are plotted as paired samples. Dotted lines represent the limit of detection. Significant differences between paired groups for each cytokine are indicated by * = $p < 0.05$, ** = $p < 0.01$, and *** = $p < 0.001$.

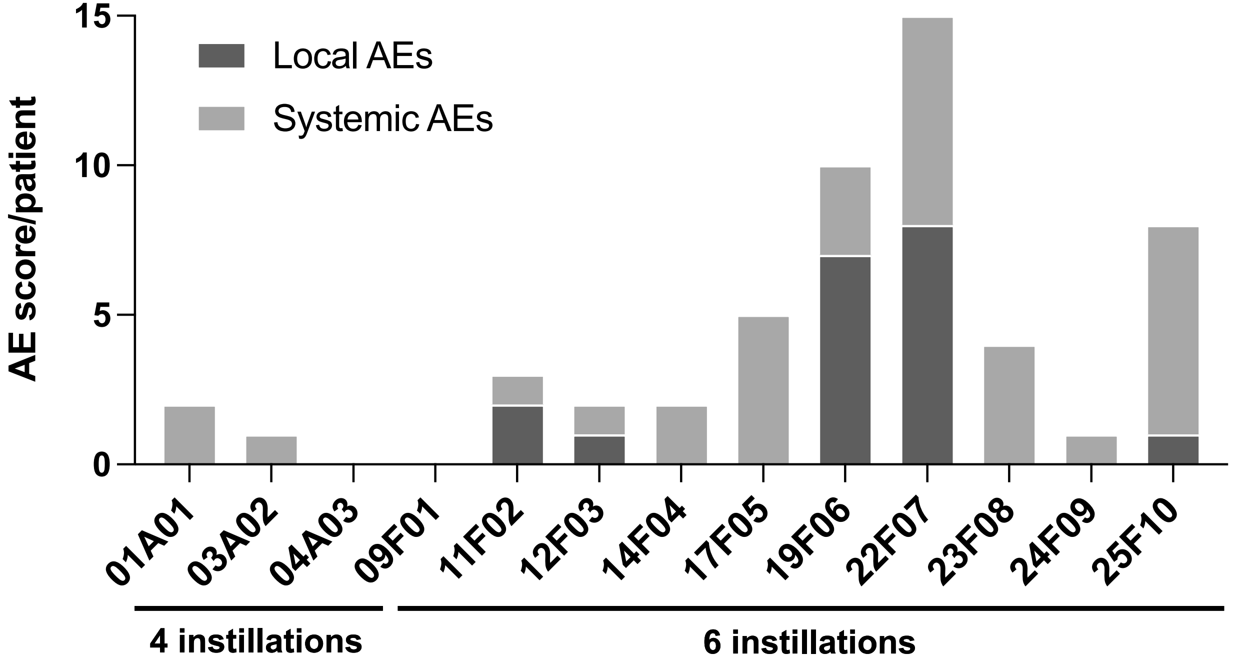

Supplementary Figure S5: Adverse event score per patient upon Ty21a treatment.

Numbers of local bladder (dark gray) or systemic (light gray) adverse events (AEs) during the 4 (group A) or 6 instillations (group F) were piled up for each patient. The AE score is the sum of the number and intensity (CTCAE 1 or 2) of AE at each visit V1 to V4 or V6. The total AE score/patient is used in Figure 1B.

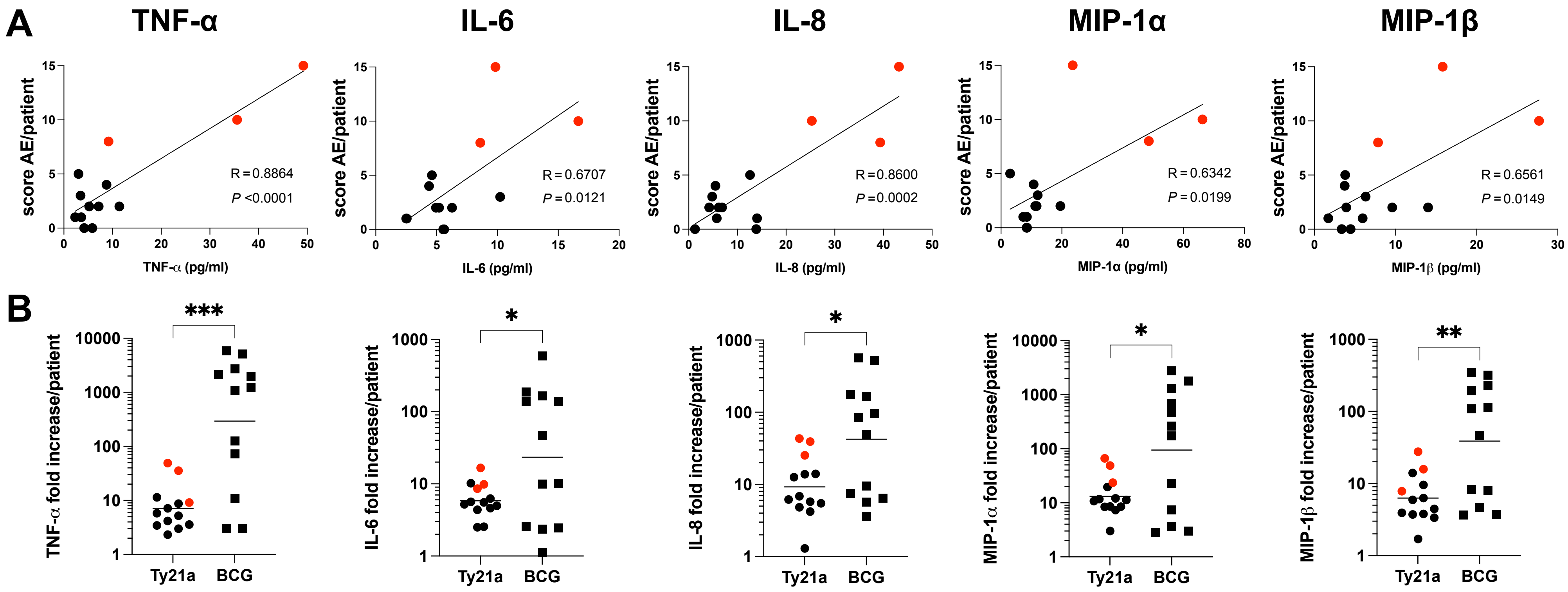

Supplementary Figure S6: TNF-α, IL-6, IL-8, MIP-1α or MIP-1β fold increases and AEs upon Ty21a or BCG treatment

A, Individual increases of urinary inflammatory cytokines (TNF-α, IL-6, IL-8, MIP1α or MIP1β) upon Ty21a treatment are plotted against the AE score (sum of the number and intensity of AE at each visit V1 to V6) of the corresponding patient. Correlation was assessed by a Pearson test, r and *P* values are indicated. **B,** Individual fold increases of each urinary inflammatory cytokine upon Ty21 or BCG treatment. Horizontal bar indicates geometric means. Groups were compared by an unpaired t-test. *: p< 0.05, ** p< 0.01 and *** p< 0.001.

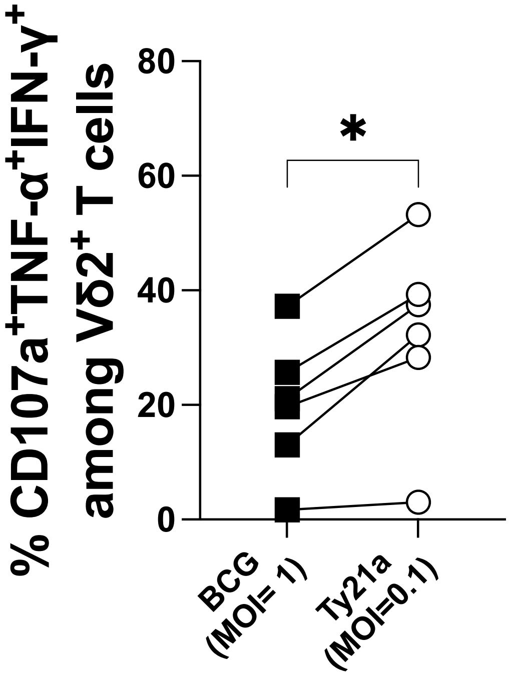

Supplementary Figure S7: Comparison of Vδ2 T-cell response upon BCG or Ty21a stimulation at different MOI

Frequency of triple positive (CD107a+TNF-α^{+}IFN-γ^{+}) Vδ2 T cells from peripheral blood mononuclear cells of health individuals, upon overnight stimulation with BCG at MOI=1 or Ty21a at MOI=0.1. Statistical significance was assessed by non-parametric paired t-test. *: $p < 0.05$.

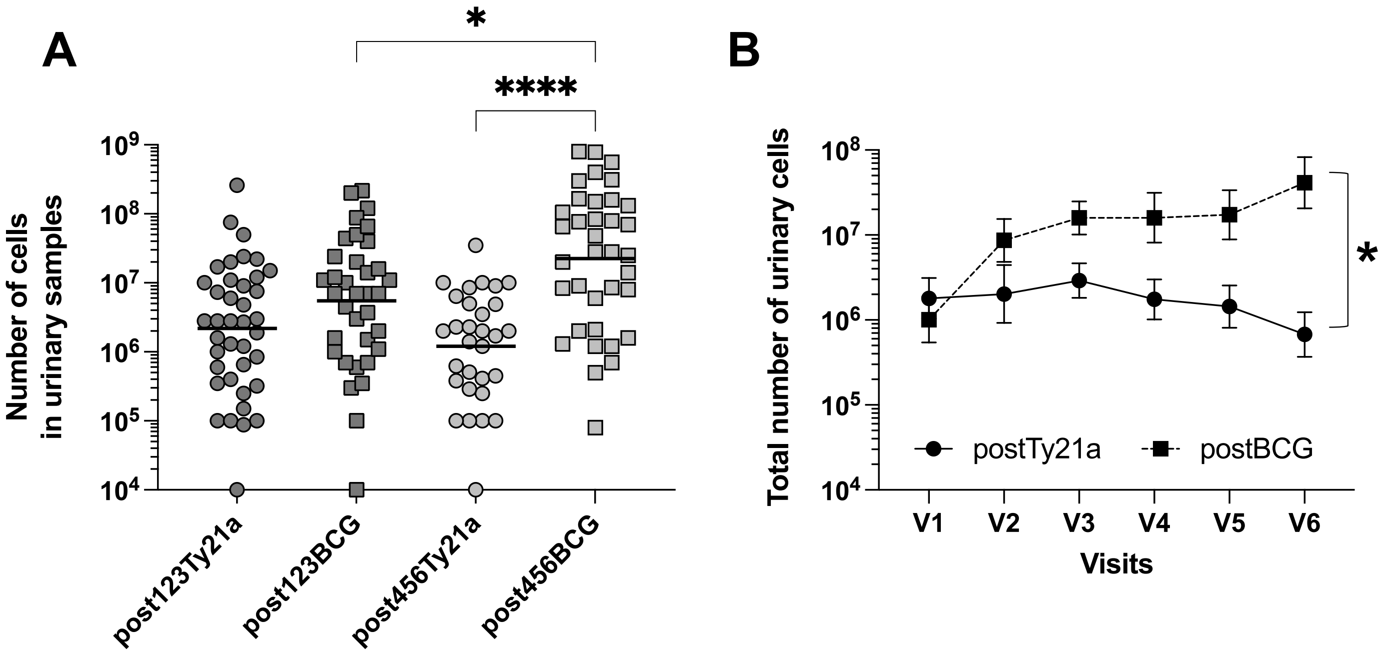

Supplementary Figure S8: Total number of urinary cells in urinary samples from Ty21a and BCG-treated patients

A, Number of urinary cells in individual patients obtained after instillations are shown as samples from post1, 2 and 3 (post123Ty21a or post123BCG) and post4, 5 and 6 instillations (post456Ty21a or post456BCG). Selected comparisons were performed using ANOVA followed by Bonferroni post-test. **B,** Number of urinary cells from Ty21a and BCG-treated patients after each instillation were recorded and geometric mean values at each visit are shown. Statistical significance was assessed by comparison of the area under the curve for each treatment using parametric unpaired t-test. *: $p< 0.05$ and **** $p< 0.0001$.
